# A Diagnostic Uncertainty in an Immunocompromised Patient: Rapidly Progressive Interstitial Lung Disease vs. Pneumocystis Pneumonia

**DOI:** 10.7759/cureus.82291

**Published:** 2025-04-15

**Authors:** Ibrahim Shamasneh, Neaam Al-Bahadili, Faustina Amable, Mohamad Rezek, Andrew McKown

**Affiliations:** 1 Internal Medicine, Piedmont Athens Regional Medical Center, AU/UGA Medical Partnership, Athens, USA; 2 Pulmonary and Critical Care Medicine, Piedmont Athens Regional Medical Center, AU/UGA Medical Partnership, Athens, USA

**Keywords:** anti-mi2 antibodies, idiopathic inflammatory myopathies, interstitial lung disease, paraneoplastic myositis, pjp pneumonia

## Abstract

Idiopathic inflammatory myopathies (IIM) are a diverse group of autoimmune disorders characterized by muscle weakness and involvement of extra-muscular organs, including the skin and lungs. The occurrence of interstitial lung disease (ILD) is considered a poor prognostic factor. While antibodies such as anti-Jo-1 and anti-MDA5 are associated with an increased risk of developing ILD, the presence of anti-Mi2 antibodies typically confers a favorable prognosis, with rare lung involvement. This case report presents a diagnostically challenging instance of a patient with positive anti-Mi2 antibodies who developed rapidly progressive ILD (RP-ILD) versus possible Pneumocystis jiroveci pneumonia (PJP).​ We present a 74-year-old Caucasian woman with a history of Stage IV adenocarcinoma with brain metastasis, complicated by vasogenic edema, treated with 12 mg of dexamethasone daily. Two weeks later, she developed proximal muscle weakness and was diagnosed with steroid-induced myopathy. Four weeks afterward, she presented with progressive shortness of breath and hypoxia, requiring high-flow nasal cannula and ICU admission. CT imaging revealed new multifocal opacities with perihilar ground-glass opacities. Review of previous investigations showed linear ground-glass opacities in the left lung and a positive anti-Mi2 autoantibody. The patient was started on intravenous steroids and intravenous immunoglobulin (IVIG) with subsequent improvement. On day five of hospitalization, serum beta-D-glucan returned elevated. Due to overlapping features with PJP, trimethoprim/sulfamethoxazole was initiated. The patient improved significantly and was later discharged on room air. Follow-up imaging six months later showed near-complete resolution. This case highlights the diagnostic complexity in critically ill, immunosuppressed patients with acute respiratory failure, where both autoimmune and infectious etiologies, such as PJP, should be considered.

## Introduction

Idiopathic inflammatory myopathies (IIM) are a diverse group of autoimmune disorders primarily characterized by muscle weakness and involvement of extra-muscular organs, notably the skin and lungs. The most prevalent subtypes are dermatomyositis and polymyositis. Interstitial lung disease (ILD) co-occurrence is a poor prognostic factor, with an estimated prevalence of 41% [1]. Autoantibodies are detected in up to 80% of IIM cases, each associated with specific phenotypic manifestations. Patients with anti-Jo-1 and anti-melanoma differentiation-associated gene 5 (anti-MDA5) antibodies, and anti-ARS antibodies more broadly, are at increased risk for developing ILD. Conversely, the presence of anti-Mi2 antibodies is generally a favorable prognostic factor, commonly manifesting with cutaneous lesions such as Gottron’s papules and heliotrope rash, but rarely associated with lung involvement [2]. We present the case of a 74-year-old Caucasian woman with progressive shortness of breath, immunosuppression from high-dose corticosteroids, and a diagnostic dilemma involving possible RP-ILD versus Pneumocystis jiroveci pneumonia (PJP).

## Case presentation

A 74-year-old Caucasian female who presented with progressive shortness of breath. She has a history of stage IV lung adenocarcinoma with brain metastasis, complicated by vasogenic edema on 12 mg dexamethasone daily started six weeks prior. Four weeks earlier, she was hospitalized with a chief complaint of bilateral proximal upper extremities weakness. After extensive workup, she was diagnosed with steroid-induced myopathy. Given the benefit that outweighs the risk, she was discharged on dexamethasone and a follow-up with physical therapy. On physical examination, she was found to be in respiratory distress, hypoxic with O_2_ saturation of 69%, with notable diffuse crackles. On neurological examination, she had upper extremities proximal muscle weakness graded as 3/5. She was initiated on 15 L/min of oxygen support through nasal cannula, and later escalated to high-flow nasal cannula with 60 L/min flow and 60% FiO_2_ to maintain her SpO_2_ saturation > 92%.

**Figure 1:**
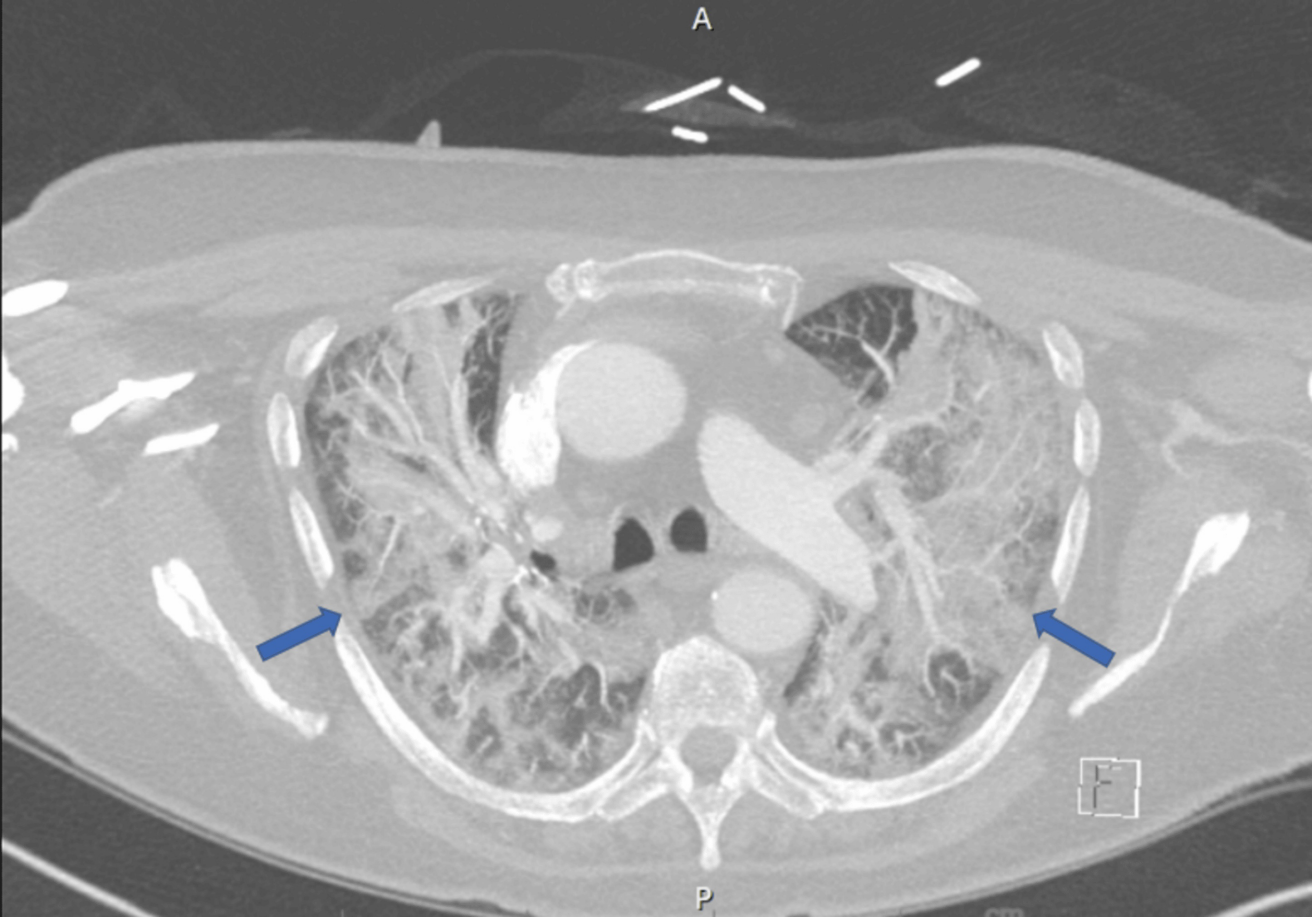
Chest CT scan shows extensive perihilar dense consolidation and ground-glass opacities with interstitial coarsening.

Initial laboratory investigations, including complete blood count and metabolic panel, were normal. A chest computed tomography (CT) scan revealed new multifocal opacities with perihilar ground-glass-like opacities, as shown in Figure 1.

Initial concern was for severe community-acquired pneumonia; thus, she was started on empirical antibiotic therapy with vancomycin, cefepime, and azithromycin, along with hydrocortisone 200 mg. She was admitted to the intensive care unit, given increasing oxygen requirements. Subsequently, respiratory culture, viral panel by polymerase chain reaction (PCR, Table 1), Legionella and Streptococcus pneumoniae urine antigen testing were all negative. Blood cultures remained negative for five days. Therefore, antibiotics were de-escalated and stopped at day three of admission.

**Table 1:** Respiratory molecular testing by polymerase chain reaction (PCR). MRSA: Methicillin-resistant Staphylococcus aureus; SARS‑CoV‑2: Severe acute respiratory syndrome coronavirus 2

Organism	Result
Adenovirus	Not Detected
Coronavirus HKU1	Not Detected
Coronavirus NL63	Not Detected
Coronavirus OC43	Not Detected
Human Metapneumovirus	Not Detected
Bordetella pertussis	Not Detected
Bordetella parapertussis	Not Detected
Chlamydia (Chlamydophila) pneumoniae	Not Detected
Mycoplasma pneumoniae	Not Detected
Human Rhinovirus/Enterovirus	Not Detected
Influenza A	Not Detected
Influenza B	Not Detected
Parainfluenza Virus 1	Not Detected
Parainfluenza Virus 2	Not Detected
Parainfluenza Virus 3	Not Detected
Parainfluenza Virus 4	Not Detected
Respiratory Syncytial Virus	Not Detected
SARS-CoV-2	Not Detected
MRSA	Not Detected

After careful review of prior imaging and work-up a month earlier, it was revealed that the patient had linear ground-glass opacities in the left lung and a positive anti-Mi2 autoantibody (level = 13, reference SI <11). This raised concerns for a paraneoplastic syndrome that includes myopathy and ILD, despite its rare association with this antibody. Bronchoscopy was also considered, but given her high oxygen requirements and our strong suspicion, it was omitted. In light of this, she was initiated on intravenous methylprednisolone 1 mg/kg daily for three days, in addition to intravenous immune globulin (IVIG) at 2 mg/kg daily for four days, started on day three of hospitalization. She had a significant improvement in her oxygen requirements.

Serum beta 1,3-D-glucan was also tested, with results that came back positive on day five of hospitalization, with levels greater than 500 pg/mL (reference <60 pg/mL). However, urine histoplasma antigen, aspergillosis, and cryptococcal were all negative. Due to overlapping features with PJP, trimethoprim/sulfamethoxazole (TMP/SMX) was initiated at a therapeutic dose on day five of hospitalization. Unfortunately, no further testing was done for PJP as the patient had significant improvement after initiating steroids and IVIG, with no further sputum production for testing.

Eventually, the patient was discharged on room air with glucocorticoids taper for rapid progressive-ILD (RP-ILD), TMP-SMX for PJP treatment, and a prearranged follow-up with pulmonary medicine.

**Figure 2:**
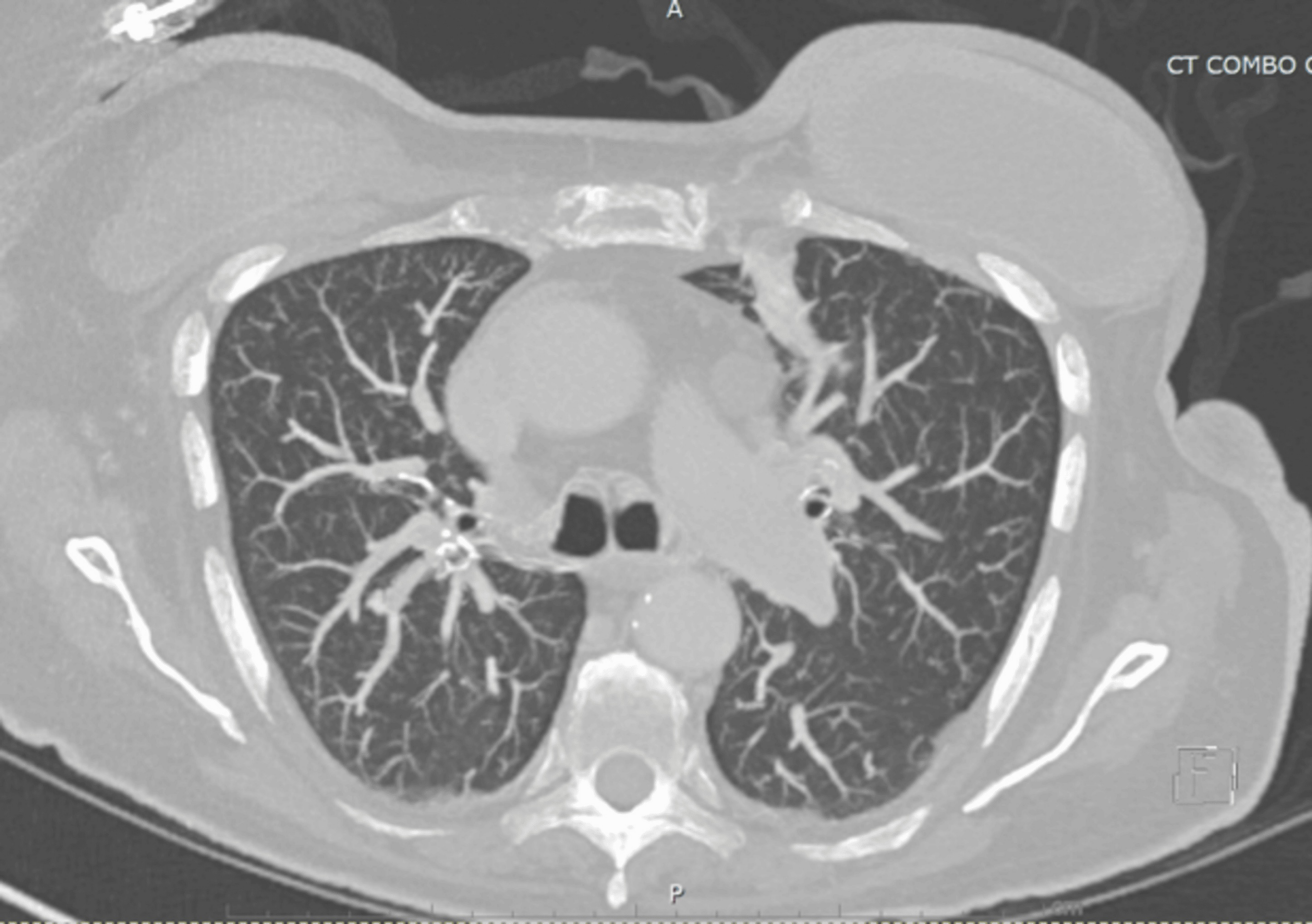
Chest CT scan showing marked improvement in consolidations six months later.

Upon follow-up, the patient remained saturating well on room air with nearly complete resolution of symptoms and lung findings on chest CT scan six months later, as shown in Figure 2.

## Discussion

IIM are a diverse group of autoimmune disorders characterized by muscle weakness and involvement of extra-muscular organs, notably the skin and lungs [1]. ILD is the hallmark of lung involvement in IIM, presenting in two primary clinical patterns: chronic ILD (defined as the presence of respiratory symptoms and/or radiological alterations for > 3 months) or RP-ILD (develops within a few weeks). The estimated prevalence of RP-ILD in dermatomyositis is approximately 8.9%, with studies suggesting a higher predisposition among Asian populations [2]. The clinical presentation of RP-ILD in dermatomyositis is similar to idiopathic ILD and includes progressive dyspnea, cough, fatigue, and weight loss. ILD presence is associated with a poorer prognosis. Although the exact cause of dermatomyositis-associated ILD remains unclear, immune-mediated inflammation with alveolar damage is believed to be the primary cause [3]. Anti-Jo-1 and anti-MDA5 antibodies are commonly associated with ILD in dermatomyositis, whereas anti-Mi2 antibodies are typically associated with skin and muscle involvement without lung involvement [2]. However, it can occur despite the rarity [4]. Our patient was severely immunosuppressed due to corticosteroids for brain metastasis-related edema. She developed acute hypoxic respiratory failure with CT evidence of diffuse pulmonary involvement. This prompted broad-spectrum antibiotic initiation, which is a common approach in critically ill patients with respiratory failure. Although anti-Mi2 antibodies were positive and imaging one month earlier showed subtle ILD, the antibody titer was only modestly elevated, and other dermatomyositis features such as rash or creatine kinase enzyme elevation were absent. The microbiological tests, including negative blood cultures, respiratory PCR panels, fungal urine and serum antigen tests, at first did not suggest an infectious etiology. Thus, antibiotics were discontinued. However, the high level of serum beta-D-glucan at day five was strongly suggestive of fungal infection, particularly PJP, which is common in immunosuppressed patients. Although no PJP-specific PCR or BAL was performed, which constitutes a limitation in this case, the clinical improvement following steroids and IVIG before TMP-SMX complicates interpretation. However, corticosteroids are also used as adjunct therapy in PJP, and we could not exclude it [5,6]. This case presents a diagnostic challenge due to overlapping features of RP-ILD and PJP. A definitive diagnosis could not be established, and it remains possible that either condition - or a combination of both - contributed to the clinical course. The presence of a positive anti-Mi2 test, despite its low titer and absence of classic features, was one of several factors considered in the differential diagnosis.

## Conclusions

This case highlights the diagnostic complexity of evaluating respiratory failure in an immunocompromized patient. Although RP-ILD was considered due to anti-Mi2 positivity and imaging, the patient’s immunosuppression and elevated beta-D-glucan were more strongly point toward PJP. Clinical improvement may be attributable to either or both treatments. This case underscores the importance of maintaining a broad differential and recognizing diagnostic uncertainty in similar presentations.
